# Six ways of failing to see (and why the differences matter)

**DOI:** 10.1177/20416695231198762

**Published:** 2023-09-27

**Authors:** Makaela Nartker, Chaz Firestone, Howard Egeth, Ian Phillips

**Affiliations:** Department of Psychological and Brain Sciences, 1466 Johns Hopkins University, Baltimore, MD, USA; Department of Psychological and Brain Sciences, 1466 Johns Hopkins University, Baltimore, MD, USA; Department of Philosophy, Johns Hopkins University, Baltimore, MD, USA; Department of Psychological and Brain Sciences, 1466 Johns Hopkins University, Baltimore, MD, USA; Department of Psychological and Brain Sciences, 1466 Johns Hopkins University, Baltimore, MD, USA; Department of Philosophy, Johns Hopkins University, Baltimore, MD, USA

**Keywords:** inattentional blindness, attention to features/objects, object recognition, perception, visual search

## Abstract

Sometimes we look but fail to see: our car keys on a cluttered desk, a repeated word in a carefully proofread email, or a motorcycle at an intersection. Wolfe and colleagues present a unifying, mechanistic framework for understanding these “Looked But Failed to See” errors, explaining how such misses arise from natural constraints on human visual processing. Here, we offer a conceptual taxonomy of six distinct ways we might be said to fail to see, and explore: how these relate to processes in Wolfe et al.’s model; how they can be distinguished experimentally; and, why the differences matter.

## Introduction

**Figure 1.**
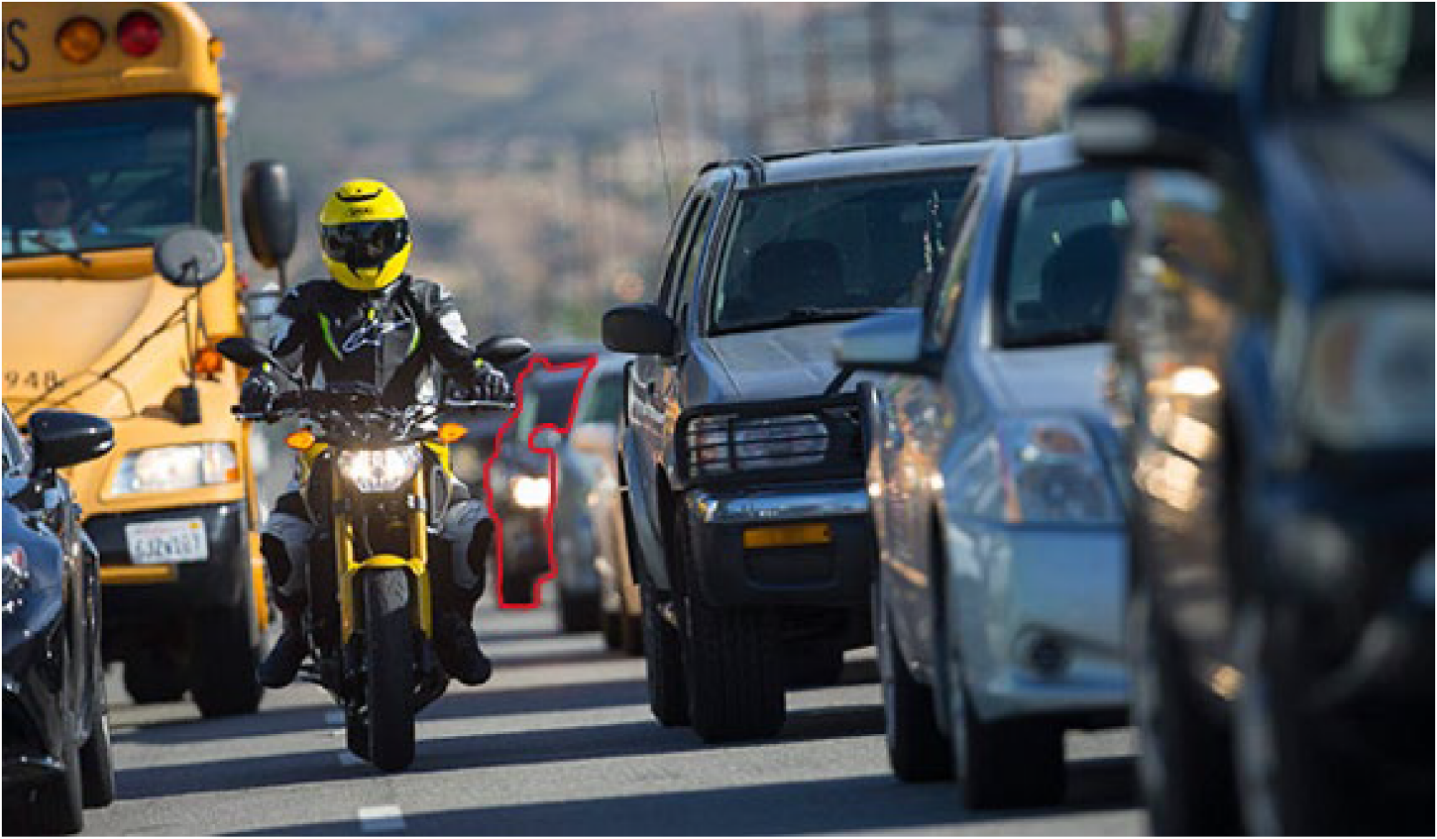
Imagine you are driving the vehicle outlined in red. There are a variety of ways that you could “look but fail to see” the motorcyclist in yellow, each of which locates the failure at a different stage of visual processing, some perceptual, some postperceptual. (a) *Selection failure*: If you do not make an eye movement near enough to the motorcycle to resolve it, you may experience only a smudge or a blur that cannot be recognized as a motorcycle. (b) *Accumulation failure*: Even if you fixate the motorcycle directly, your thresholds for detection, identification, or recognition may be too conservative (e.g., because a motorcyclist in between lanes is a relatively rare occurrence, or because the visual input to any of these processes was already degraded by failures in selection); thus, you may not *detect* the motorcycle, or you may fail to recognize its categorical membership (“motorcycle”) and so not respond to it appropriately. (c) *Memory failure*: As you fixate multiple times around this scene, later fixations might degrade or interfere with the earlier encoding from your initial fixation—meaning that you might *see, resolve,* and even *detect* the motorcyclist, but fail to take it into account when changing lanes many seconds later.

We often fail to respond to stimuli right before our eyes, such as a motorcyclist at an intersection or a typo in a manuscript (see Figure 1). Wolfe et al. (2022) propose that these “Looked But Failed to See” (LBFTS) errors reflect ordinary constraints on visual processing, and offer a common mechanistic framework. Their model posits processes of *selection* (privileging certain items), *information accumulation* (gathering evidence for an object property), and *termination* or *quitting* (deciding to stop accumulating evidence, or ceasing the task altogether). These processes also represent algorithmic break points responsible for LBFTS errors. For example, Wolfe et al. note that misses might arise at the *selection* stage if one's fixation lands too far from a target to select it, or if the visual field around the object is too crowded to resolve its features. Likewise, misses might arise at the *accumulation* stage if one does not attend long enough to gather sufficient evidence to reach thresholds for object identification or recognition.

Wolfe and colleagues’ insightful discussion illuminates the mechanisms that produce LBFTS errors. It also raises a deep conceptual question: What is it to “fail to see”? Here, we distinguish six possible answers, from two broad categories: perceptual errors (roughly, those occurring between sensory registration on the retina and the generation of a conscious percept), and postperceptual errors (which arise after visual processing is complete and are attributable to processes such as memory, judgment, and verbal report). We further explore where each type of LBFTS error fits in Wolfe et al.'s model, and why the differences matter.

## Postperceptual Errors

Wolfe et al. define LBFTS errors as failures “to respond successfully to stimuli that are unambiguously visible.” Such failures can occur for broadly postperceptual reasons.

1. Observers may see a stimulus momentarily but fail to encode it in explicit *memory*. This failure is central to “inattentional amnesia” accounts of inattentional blindness (Wolfe, 1999). Evidence for inattentional amnesia comes from studies of perceptual grouping without attention. For instance, Moore and Egeth (1997; replicated in Wood & Simons, 2019) found that line-length judgments were affected by Ponzo and Müller-Lyer illusion cues, suggesting that such cues were in some sense seen and processed, even when observers failed to report them. Memory failures show that sensory evidence must be encoded and maintained to inform later judgments.
2. Observers might fail to respond because they lack *confidence* in what they saw. In detection or drift-diffusion models, this can be captured by a conservative response criterion influenced by numerous factors, including subjects’ priors and pay-off matrices (e.g., the penalty for false alarms). Notoriously, participants exhibit a pervasive under-confidence bias in perceptual tasks, especially with weak signals, performing better in discrimination tasks than their confidence suggests (Björkman et al., 1993). Such conservative responding is a familiar concern in many different contexts from blindsight (Campion et al., 1983; Phillips, 2021) through semantic priming (Holender, 1986) to change blindness (Hollingworth et al., 2001; Mitroff et al., 2004). These biases must be considered regarding the thresholds in Wolfe et al.'s model.
3. Consider the thought that you *must* have seen something but failed to *notice* it—the keys you had lost which turn out to be right where you had been looking, a repeated word in a sentence, etc. It is controversial whether conscious seeing can truly occur without noticing (or recognizing, identifying, etc.) of any kind (Dretske, 1979; Dennett, 1994)—a possibility closely tied to the vexed question of whether phenomenal consciousness overflows cognitive access (Block, 1995; Phillips, 2018). However, if perception can occur without noticing, and noticing precedes judgment, failures to notice would constitute a third class of postperceptual LBFTS error. In Wolfe et al.'s framework, these distinctions might be modeled by distinguishing additional evidence *thresholds* within the same accumulation process*,* or by altogether separate *accumulators* and *thresholds*.

## Perceptual Errors

Many LBFTS errors do occur for broadly perceptual reasons.

4. Vision may become *degraded* in myriad ways such that it does not represent the world in crisp and complete detail. Degradation can yield LBFTS errors without a *complete* failure to see. Wolfe et al. note that some LBFTS errors result from limitations in visual acuity preventing us from resolving object details away from fixation (the “functional” or “useful” field of view); in other words, if we do not fixate near the motorcycle, we may detect something where the motorcycle is but not *recognize* it as such. Likewise, in crowding, observers may fail to recognize a detected stimulus due to limitations on feature integration (Levi, 2008). Similarly, *inattention* may degrade vision by making only low-level visual features available. Consequently, a passing gorilla might appear only as an unrecognizable dark smudge—so-called “inattentional agnosia” (Simons, 2000). Wolfe et al. explore another species of degradation in which vision only represents summary statistics without selective attention (Cohen et al., 2016). Again, this may cause observers to miss specific targets. This said, Wolfe et al. claim that we do not experience a statistical summary but rather the most likely state of the world consistent with such statistics. On this view, LBFTS errors may arise from misrepresenting the world as being in a likely state, excluding an unlikely target.
5. Visual processing can sometimes fail to generate *conscious* perception. The nature and even existence of unconscious perception are controversial (Peters et al., 2017), but a consensus view is that perceptual processing occurs outside awareness and so may occur even when a subject consciously misses a target.
6. Finally, some LBFTS errors may result from a *total* failure to see, consciously or otherwise. This will occur if the very early processing of stimuli is prevented or suppressed, leaving the subject truly blind. Again, limits on our visual system may be responsible—e.g., eye movements create massive retinal blur and demand saccadic suppression, leaving us unable to detect concurrent changes, as in saccade-contingent change blindness (Grimes, 1996). Such cases may extend the term “LBFTS error” beyond what is typical in the literature, but nonetheless represent an extreme case of stimuli we may look at and yet fail to see.

## Distinguishing the Six Ways We Fail to See

Our taxonomy is not intended as exhaustive: There may be other ways we fail to see and other important distinctions to draw. Moreover, we recognize that there will be disagreement as to exactly how and where to draw the line between perceptual and postperceptual errors (for a recent discussion, see Block, 2023). Nonetheless, it raises an important challenge: How can we experimentally distinguish among the six errors we identify? And what difference does doing so make?

One powerful approach exploits signal detection theory (Green & Swets, 1966) to distinguish between LBFTS errors resulting from a lack of perceptual sensitivity (i.e., 4–6), and errors resulting from conservative decision bias (i.e., 2). To achieve this, our studies (Nartker et al., 2021) adapt traditional IB paradigms by including absent trials. Our findings suggest that LBFTS errors are partly due to under-confidence. Observers adopt conservative response criteria when reporting awareness of unexpected stimuli (suggesting postperceptual under-confidence). Consistent with this, observers who claim not to have seen anything unusual (i.e., respond “no” under yes/no questioning about noticing any unexpected stimuli) perform above chance when asked whether the stimulus was on the left or right, or was blue or red. Of course, one reason observers likely adopt conservative criteria is that inattention has degraded their perceptual sensitivity, indicating a second interacting source of LBFTS errors in IB. In terms of Wolfe et al.'s model, our findings suggest *both* a slow rate of evidence accumulation (due to perceptual degradation) and a high bar for such evidence to reach (due to conservative decision thresholds).

Wu and Wolfe (2018) have their own methodology for revealing that subjects see more than they report: They asked subjects to track up to 32 objects and report where a randomly queried object was at the end of each trial. As set sizes increased to more than four objects, subjects increasingly failed to locate the queried object on their first click. However, when allowed to continue clicking, Wu and Wolfe found that subjects located the target with far fewer clicks on average than random guessing would have required. In short, subjects had more information about each object's location than single responses revealed.

A final approach (cited by Wolfe et al.) analyzes eye movements to isolate the type of LBFTS error. For instance, using a driving simulator, Robbins et al. (2019) found that drivers deciding whether to pull out sometimes failed to report an oncoming motorcycle even after fixating it, and moreover that such misses were correlated with the number of head movements made between fixation and report. These results suggest that the motorcycle may have initially been seen, but that subsequent information interfered with its encoding and/or retrieval. In terms of Wolfe et al.'s model, we can think of such errors as due to information loss prior to a decision, meaning that acquired evidence no longer meets the threshold.

## Why the Differences Matter

Understanding exactly where LBFTS errors originate may help identify new ways to reduce their prevalence and mitigate their impact, with important real-world implications for training, design, and strategic intervention. For instance, if an LBFTS error is due to subjects routinely setting too conservative a criterion, we can manipulate criteria through training, whether by explicit instruction (Morgan et al., 2012) or modifying implicit payoffs. Similarly, if an LBFTS error is due to memory interference (Robbins et al., 2019), we can overcome it using explicit rehearsal or cue-based retrieval strategies. In contrast, if an LBFTS error is perceptual, we must instead determine what the relevant limitation is and seek perceptual or environmental solutions—for instance, by designing safety clothing and signage with relevant perceptual limitations in mind, or by optimizing search strategies so that limited perceptual resources are most effectively deployed (Dzeng et al., 2016). There are also less obvious implications. For instance, if Wolfe et al. are right that under nonselective processing we experience the most likely state of the world consistent with a scene's summary statistics, we should consider empirically determined perceptual priors when locating features such as pedestrian crossings and walkways.
